# Reliability of a Constant Sentinel Vein to Aid in Hamstring Graft Harvesting for Anterior Cruciate Ligament Reconstruction

**DOI:** 10.7759/cureus.84109

**Published:** 2025-05-14

**Authors:** Nagarjuna C.R, Yusuf Omar Qalib, A.V. Gurava Reddy

**Affiliations:** 1 Orthopaedics, Kims Hospitals, Hyderabad, IND; 2 Orthopaedics, Sunshine Bone and Joint Institute, KIMS-SUNSHINE Hospitals, Hyderabad, IND

**Keywords:** acl reconstruction, constant sentinel vein, hamstring graft harvesting, landmark techniques, pes anserinus

## Abstract

The presence of a constant sentinel vein (CSV) as an anatomical landmark improves the accuracy and efficiency of hamstring tendon harvesting during anterior cruciate ligament (ACL) reconstruction. We present a technique for using a branch of the inferior medial geniculate vein as an anatomical landmark. The CSV, which is consistently located 1-2 mm below the epidermis, serves as a reliable guide to localize the semitendinosus and gracilis tendons. Through the identification of reliable anatomical landmarks, surgeons may enhance their ability to accurately localize and harvest the hamstring tendons, potentially minimizing tissue trauma, reducing incision size, procedure time, risk of neurovascular injuries, and improving overall surgical outcomes.

## Introduction

Anterior cruciate ligament (ACL) reconstruction is among the most commonly performed orthopaedic procedures for sports-related injuries worldwide. Hamstring tendon autografts are often regarded as the preferred choice by surgeons, attributed to their advantageous biomechanical properties and lower incidence of donor-site morbidity compared to patellar tendon grafts [1,2]. However, hamstring graft harvesting presents technical challenges such as inadvertent damage to adjacent neurovascular structures (e.g., the infrapatellar branch of the saphenous nerve), premature tendon amputation, and graft size variability, all of which can compromise surgical outcomes [3,4]. Conventional methods that depend on osseous landmarks, such as the tibial tubercle or medial joint line, often require larger incisions and more extensive dissections, which can increase the likelihood of postoperative complications [5]. To address these challenges, surgeons have explored alternative techniques for hamstring graft harvesting that aim to minimize tissue trauma and improve precision. This study introduces a standardized technique for hamstring harvest using the CSV as a reliable anatomical guide. This approach potentially offers several advantages, including smaller incisions, reduced risk of neurovascular injury, and more accurate graft sizing, which could ultimately lead to improved patient outcomes and faster recovery.

## Technical report

Surgical technique for graft harvest

In arthroscopic-assisted knee procedures, patients were positioned supine on the operating table with the operative knee flexed to 90° and supported by a leg holder. A well-cushioned pneumatic high thigh tourniquet was applied to the proximal thigh, and spinal anaesthesia was administered. The limb was then prepared and draped following conventional sterile protocols. Primary anatomical landmarks, including the patellar tendon, tibial tubercle, medial and lateral joint lines, and the insertion of the pes anserinus, were delineated with a surgical marker.

**Figure 1:**
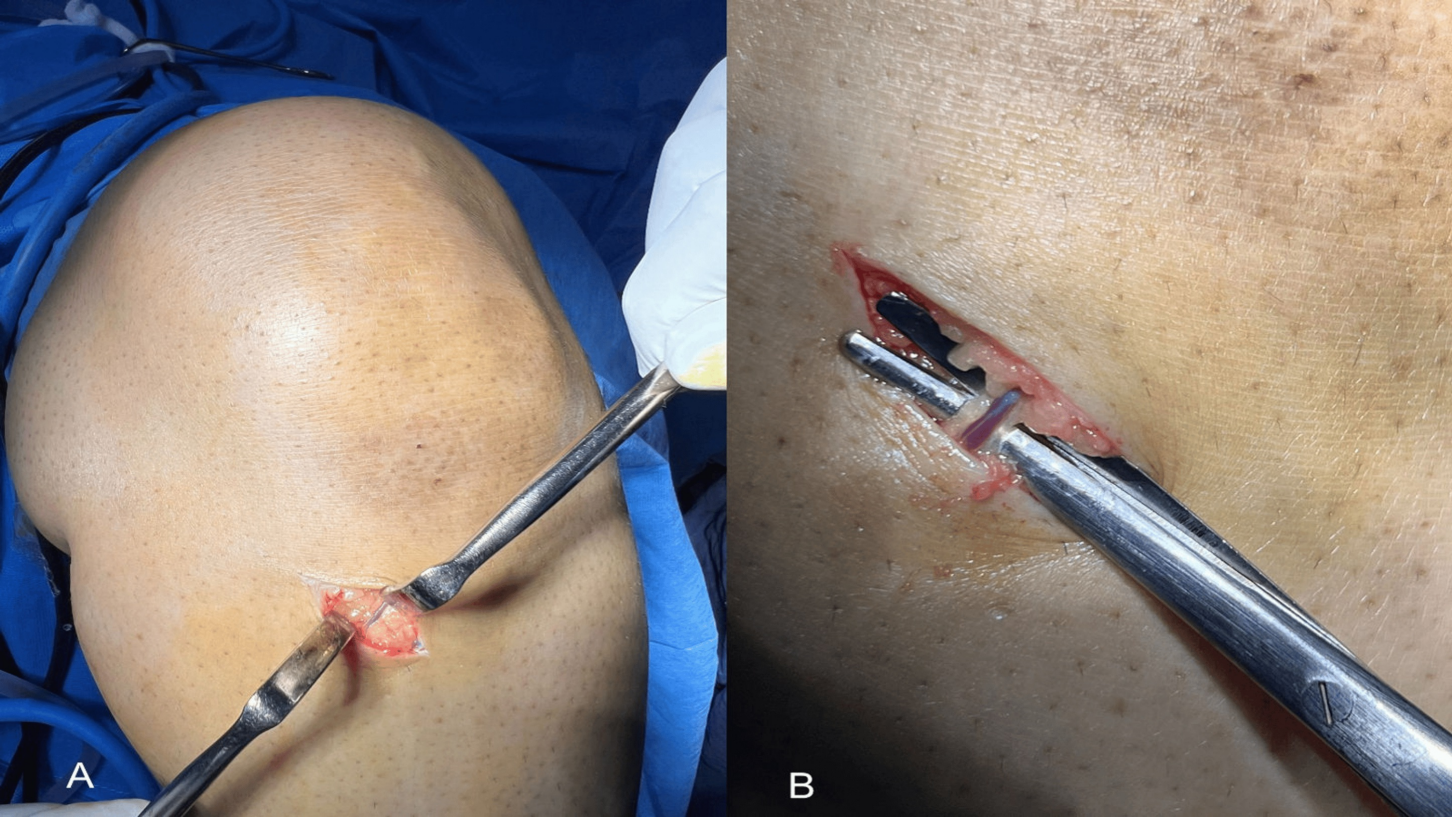
Using an oblique incision in the left knee, the constant sentinel vein was identified beneath the skin at the upper border of the pes tendons, approximately one fingerbreadth below and one fingerbreadth medial to the lower margin of the tibial tubercle (1A). Magnified view demonstrating the constant sentinel vein indicated by surgical scissors (1B)

A 2 cm oblique incision is performed, centered over the anticipated pes anserinus insertion, approximately one fingerbreadth below and one fingerbreadth medial to the lower margin of the tibial tubercle (Figure 1). Following the skin incision, a CSV, which is a branch of the inferior medial geniculate vein, can typically be observed running 1-2 mm beneath the epidermis. Upon careful dissection of the subcutaneous tissue, a branch of the inferior medial genicular artery (bIMGA) is subsequently identified deep to this vein [6]. Based on our experience, this vein is constantly present in all cases involving hamstring grafts and serves as a reliable anatomical landmark, enabling surgeons to precisely identify and harvest the hamstring tendons. Utilizing this vascular reference point allows for the straightforward identification and sequential harvesting of the gracilis and semitendinosus tendons. This process is conducted only after ensuring that the tendons are thoroughly freed from all fascial bands, in accordance with standard ACL reconstruction protocols. Using this approach, the success rate was 100%, with no associated complications observed.

## Discussion

The identification of a CSV as a viable anatomical landmark for hamstring tendon harvesting provides a solution to enduring challenges related to graft harvesting. This technique leverages the CSV's present anatomical association with the pes anserinus, providing a dependable reference for locating the semitendinosus and gracilis tendons while reducing soft-tissue dissection. Several external landmarks and techniques aid in identifying the hamstring tendons, with the tibial tubercle being the most commonly used reference for determining the skin incision site. Palpation of the pes tendons also provides additional guidance; however, this approach can occasionally make it difficult to locate the pes insertion, particularly for less experienced surgeons or in individuals with substantial subcutaneous tissue over the pes anserinus [7,8]. The CSV technique presents potential advantages in terms of precision and efficiency in hamstring tendon harvesting procedures. By employing this anatomical landmark, surgeons may reduce the risk of iatrogenic injury to adjacent structures and decrease surgical duration. Moreover, the CSV approach could be particularly beneficial for novice surgeons, offering a more reliable method for locating the target tendons compared to traditional external landmark techniques.

Our approach is in agreement with prior anatomical studies, which emphasize the critical role of vascular landmarks in enhancing the precision of surgical procedures during ACL reconstruction [7,9]. This minimizes dependence on inconsistent osseous landmarks and unguided fascial dissection, which have traditionally been linked to prolonged operative times and a heightened risk of iatrogenic injury [10]. To the best of our knowledge, this CSV, which is a branch of the inferior medial geniculate vein, has not been previously described or named in existing literature. In light of its reliability as an anatomical landmark during the harvest of the hamstring tendons, we propose the nomenclature Vena Nagalib's Vigilis Anserina (VNVA) to refer to this structure. Future research should prioritize large-scale, multicenter trials to validate the CSV’s universal applicability, particularly in patients with prior knee surgery or obesity, where anatomical distortion may challenge reproducibility.

## Conclusions

The CSV-guided approach to an anatomical landmark constitutes a notable advancement in hamstring graft harvesting, integrating anatomical precision with minimally invasive principles. By minimizing the incision size, reducing operative time, and mitigating neurovascular risks, this method aligns with contemporary orthopaedic priorities of patient safety and procedural efficiency. Notably, using this method, a 100% success rate was achieved with no associated complications observed.
